# Subthreshold laser therapy with a standardized macular treatment pattern in chronic central serous chorioretinopathy

**DOI:** 10.1007/s00417-021-05256-3

**Published:** 2021-06-10

**Authors:** Benedikt Schworm, Jakob Siedlecki, Leonie F. Keidel, Tina R. Herold, Nikolaus Luft, Siegfried G. Priglinger

**Affiliations:** Department of Ophthalmology, Ludwig-Maximilians-University Munich, Mathildenstrasse 8, 80336 Munich, Germany

**Keywords:** Central serous chorioretinopathy, Chronic central serous chorioretinopathy, Pachychoroid, Laser treatment, Nondamaging laser, Subthreshold laser, Macular pattern

## Abstract

**Purpose:**

There is an ongoing controversial debate about the effectiveness of laser treatments in chronic central serous chorioretinopathy (cCSC). We performed a prospective non-randomized interventional study to learn about the effects of a subthreshold laser treatment (Topcon Endpoint Management™, Topcon Healthcare Inc., Tokyo, Japan) in patients with cCSC.

**Methods:**

Patients with cCSC and a minimum symptom duration of 4 months were included and treated with a standardized laser pattern covering the macular area. Retreatment was performed every 3 months if persistent subretinal fluid was observed. The primary endpoint was resolution of subretinal fluid at 6 months. Further outcome parameters included best corrected visual acuity, microperimetry, central macular and subfoveal choroidal thickness.

**Results:**

A total of 42 eyes of 39 patients were included. Mean patient age was 48 ± 10.6 years (range 25–67). Mean symptomatic time before inclusion into the study was 134 ± 133.4 weeks (16–518). Before inclusion, 78.6% of the patients had failed to resolve subretinal fluid under mineralocorticoid receptor antagonists and 14.3% had a recurrence after half-dose photodynamic therapy. Complete resolution of subretinal fluid was observed in 42.9% at 6 months and in 53.8% at 12 months after baseline. Central retinal thickness decreased from 398 ± 135 µm to 291 ± 68 µm (p < 0.001), subfoveal choroidal thickness changed slightly (430 ± 116 µm to 419 ± 113 µm, p = 0.026), microperimetry-derived macular function improved by 19.1 ± 4.7 dB to 21.3 ± 4.8 dB (p = 0.008) and mean BCVA improved by 4.9 ± 8.6 ETDRS letters (p < 0.001).

**Conclusion:**

The results show that the investigated laser treatment is effective in reducing subretinal fluid and leads to an improvement of functional parameters.


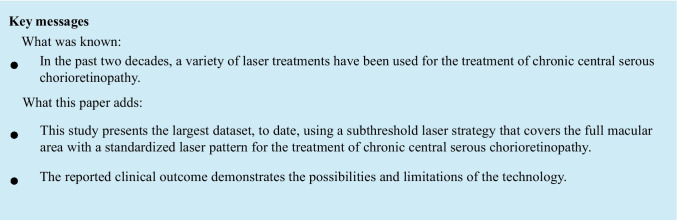


## Introduction

Central serous chorioretinopathy (CSC) is a retinal disease typically affecting the macular area with subretinal accumulations of fluid leading to a serous detachment of the neurosensory retina. Men are more often affected than women and first episodes usually occur in middle aged individuals [1].

Acute episodes of CSC show a high rate of spontaneous resolution (up to 84% in 6 months) [2]. However, the disease has a strong tendency to become chronic manifesting in frequent recurrence or persistent subretinal fluid which can then lead to sight-threatening long-term complications such as progressive damage of the outer retina, disruptions of the retinal pigment epithelium and secondary choroidal neovascularization [3].

While acute episodes can be observed whether spontaneous resolution occurs in the first 2 to 4 months [4], cCSC requires treatment in order to avoid the long-term complications listed above. Thus, a variety of treatment options have been considered in the past, the most widely applied approaches being verteporfin photodynamic therapy (PDT), laser treatments and the oral administration of mineralocorticoid-receptor (MR) antagonists. The search for the most effective therapy has increasingly become focus of clinical trials within the last decade.

Pharmacological treatment with oral administration of mineralocorticoid-receptor antagonists which are actually prescribed for the treatment of heart failure has been discussed with optimism [5–7]. Unfortunately, in a placebo-controlled randomized trial, the widely used agent eplerenone was not superior to placebo in terms of improving best corrected visual acuity [8].

Various laser treatments usually targeting the area of leakage on fluorescein angiography have been considered. Conventional thermal laser coagulation of leakage areas leads to retinal damage and therefore is rather seen as of historical relevance. To avoid tissue damage as a consequence of photocoagulation in the macular area, several technologies have been developed to reduce laser damage while trying to maintain therapeutic effects. A widely implemented technology is called “subthreshold micropulse laser” (SML) and uses a pulsed laser beam that delivers a burst of very short pulses (around 0.1 ms duration) over the 100 ms to 300 ms pulse duration [9]. For treatments at the posterior pole, the average power is set below a clinically detectable tissue damage threshold which coined the term “subthreshold” for these treatments. SML showed promising results for the treatment of cCSC in clinical trials [10, 11]. Nevertheless, SML was inferior to half-dose photodynamic therapy (hd-PDT) with verteporfin in a randomized-controlled multicenter trial [12].

While SML treatments are usually targeted at the site of active leakage, the laser strategy in this study was to treat the entire macular area within a radius of 3000 µm with a standardized pattern. In order to provide a laser therapy that is within the therapeutic window and at the same time “non-damaging”, a standardized titration procedure as well as an algorithm called Endpoint Management™ (EpM) with the PASCAL® Synthesis™ (Topcon Healthcare Inc., Tokyo, Japan) that adjusts the laser power for treatment was employed.

The purpose of this study was to clinically evaluate the application of a laser therapy with EpM in patients with cCSC.

## Methods

### Study design and ethics approval

This trial was a single center, investigator-initiated, prospective interventional trial. The study adhered to the tenets of the Declaration of Helsinki in its latest version (October 2013) and was approved by the ethics committee of the Ludwig Maximilian University Munich (identifier 17–879). All patients provided written informed consent before entering the study. The study was registered in the German clinical trials registry (International Clinical Trials Registry Platform (ICTRP) Identifier DRKS00014299).

### Participants

Patients with cCSC were recruited from outpatient clinics of the Department of Ophthalmology, University Hospital of Munich, LMU Munich. Inclusion criteria were: age of 18 years or older, visual acuity score of 35 or better on early treatment diabetic retinopathy study (ETDRS) charts and the diagnosis of cCSC. Imaging features of cCSC that were mandatory for inclusion were fovea-involving subretinal fluid persisting for more than 4 months [13] and mottling of the retinal pigment epithelium (RPE) on optical coherence tomography (OCT), active leakage on fluorescein angiography (FA) including hot spots or granular hyperfluorescence and diffuse hyperfluorescent changes on indocyanine green angiography (ICG) [4]. The diagnosis of cCSC needed to be confirmed by a second retinal specialist according to the above-mentioned criteria. Exclusion criteria were: Any other macular disease that could lead to subretinal fluid (such as vitreomacular traction syndrome, epiretinal membranes, age-related macular degeneration, diabetic retinopathy, choroidal neovascularization (CNV), uveitis), ocular surgery within 6 months preceding the study, previous intravitreal injections of anti-VEGF, previous macular laser treatment or photodynamic therapy within 3 months preceding the study, present ocular infection or clinically significant inflammation, kidney failure or dialysis, pregnancy or currently breast-feeding, allergy to fluorescein, history of systemic or topical corticosteroid use within the last 6 months, planned intraocular surgery within 12 months. The exclusion criteria were reevaluated at each study visit before continuing the study. During the study period, the participants attended a total of seven visits, organized as: baseline visit (including treatment) and 1, 2, 3, 6, 9 and 12 months after baseline. At each visit, a complete ophthalmological examination was performed as well as functional testing and multimodal imaging as described below.

### Functional testing

Best corrected visual acuity (BCVA) was assessed using Early Treatment Diabetic Retinopathy Study (ETDRS) charts in a standardized protocol. Refraction was reevaluated at each study visit. Retinal sensitivity was assessed using the microperimetry device “Macular Integrity Assessment” (MAIA, CenterVue Inc., Padova, Italy).

### Multimodal imaging

Multimodal imaging included enhanced depth (EDI) spectral domain optical coherence tomography (SD-OCT), blue-autofluorescence (BAF) and near-infrared (NIR) confocal laser scanning ophthalmoscopy (CSLO) in every eye at each visit (Spectralis® HRA + OCT, Heidelberg Engineering GmbH, Heidelberg, Germany). A fluorescein (FA) and indocyanine green (ICG) angiography (on Spectralis®) was performed at baseline at the final visit. OCT angiography (Cirrus 5000®, Carl Zeiss Meditec AG, Jena, Germany) was performed in every eye at each visit. Additionally, an ultra-widefield fundus image (UWF, California®, Optos Inc., Marlborough, USA) of both eyes was obtained at every visit.

### Laser treatment

Pupillary dilation was achieved by application of both tropicamide eyedrops (Mydriaticum Stulln® UD, Pharma Stulln GmbH, Stulln, Germany) and phenylephrine (Neosynephrin-POS® 5%, Ursapharm Arzneimittel GmbH, Saarbruecken, Germany) eyedrops. After topical corneal anaestesia with oxybuprocain-eyedrops (Conjuncain®, Bausch + Lomb Inc., Berlin, Germany) a contact lens (Volk Area Centralis®, Volk Optical Inc., Mentor, Ohio, USA) was placed on the eye with 1% methylcellulose as contact gel. Laser treatment was performed using the Endpoint Management™ (EpM) with the PASCAL® Synthesis™ (Topcon Healthcare Inc., Tokyo, Japan) with a wavelength of 577 nm according to a previously published protocol [14]: The treatment begins with a titration by applying single laser spots outside the vascular arcs. The titration power is then raised in steps of 10 mW until a barely visible lesion evolves. A barely visible lesion is defined as a slight greying of the retina 3 s after application (energy 100–200 mW, pulse duration 15 ms). This energy is set as 100% on the EpM scale. Then, the energy is reduced to 30% on the EpM scale for the actual treatment. The laser pattern used for treatment was a “macular grid pattern” with a spot size of 200 µm, a spot spacing of 0.25 × and 400 spots in total. The pattern is circular and covers the macular area with an outer radius of 3000 µm and with an inner radius of 500 µm which allows sparing of the fovea. During application, the pattern is divided into eight “octants” of 50 spots. Each octant is applied by a single activation of the foot pedal. During treatment, the patient was instructed to fixate a central fixation spot and the treating physician observed the fixation via the slitlamp binocular. At the first treatment, one laser spot at the outer edge of each octant was applied with the full titration energy to produce a so-called landmark spot. The landmark spots were visible at month 1 as hyperautofluorescent spots on BAF imaging and then gradually disappeared over the next months by healing processes of the RPE. In cases, where landmark spots were not visible at the control visits, the titration process was repeated at the next treatment in order to avoid undertreatment. The number of all applied laser spots ranged between 400 and 420 spots, depending on the number of titration spots.

### Retreatment

A retreatment was performed every 3 months in case of persistent subretinal fluid in the macular area (within 3000 µm radius centered to the fovea). The laser settings were the same as in the first treatment. In cases where no landmarks were visible on the NIR image at month 1, the laser energy in the titration mode was raised by 10 mW to avoid undertreatment. In case of resolved subretinal fluid, no retreatment was performed.

### Outcome measurements

Primary outcome was the absence of subfoveal subretinal fluid as assessed on OCT after 6 months. The mean change in central macular thickness (CMT) was assessed by comparing the measurements of the individual study visits with the baseline measurement. CMT was recorded from the central 1-mm-diameter circle of the ETDRS-grid in the thickness map generated by the Spectralis Software (Heidelberg Eye Explorer 1.9.11.0, Heidelberg Engineering GmbH, Heidelberg, Germany). The automatic segmentation of the retinal layers was manually corrected when necessary. Secondary outcome measures included change in BCVA, change in retinal sensitivity (assessed with MAIA), percentage of complete resolution of subretinal fluid and mean change in subfoveal choroidal thickness (SFCT). SFCT was assessed by manually measuring the distance between the Bruch’s membrane interface and the choroidal–scleral interface under the center of the foveolar depression on enhanced depth imaging OCT images in the 1:1 µm setting. All measurements were performed by two independent graders (BS, LK). In cases of disagreement, a third grader (JS) was consulted. For subgroup analysis, “Early Response” was defined as a decrease in CMT by > 15% 3 months after the baseline treatment (i.e. one laser treatment). All patients with persistent subretinal fluid (pSRF) at the last study visit were defined as “nonresponders”.

### Statistical analysis

All data were gathered in Microsoft Excel spreadsheets (Version 16.23 for Mac; Microsoft Corporation, Redmond, WA, USA). Statistical analysis was performed in SPSS Statistics 25 (IBM Germany GmbH, Ehningen, Germany). The level to indicate statistical significance was defined as p < 0.05. The Shapiro–Wilk and Kolmogorov–Smirnov tests were employed to test for normal distribution. Statistical analyses of intra-group differences were performed using the dependent two-tailed Student t-test and the Wilcoxon signed rank test. A repeated measures ANOVA test was used to compensate for multiple testing, if applicable. Pearson’s correlation coefficient was used to test associations of dependent and independent variables. The analysis was conducted based on a modified intention-to-treat approach. Consequently, all assessed data was analysed after inclusion of a subject, regardless of protocol deviations or loss to follow-up. Graphs were plotted in Microsoft Excel and/or SPSS Statistics Version 25.

## Results

### Baseline demographics

In total, 54 eyes were screened for eligibility. Of these, 8 eyes were excluded because imaging revealed presence of secondary CNV. Three eyes were excluded because of other retinal diseases besides CSC and one patient was excluded because of use of steroid medication for other than ocular disease. After applying the exclusion criteria, a total of 42 eyes of 39 patients were included in this study of which 7 (17.9%) were female and 32 (82.1%) were male patients. Mean patient age was 48 ± 10.4 years (range 25–67). Mean symptomatic time before inclusion into the study was 134 ± 133.4 weeks (16–518). Before inclusion into the study, 78.6% (n = 33) of the patients failed to resolve subretinal fluid under oral mineralocorticoid receptor antagonists and 14.3% (n = 6) had a recurrence of subretinal fluid after half-fluence photodynamic therapy (Table 1).

**Table 1:** Baseline data. Data are presented as no. (%) or mean ± standard deviation (range). MR = mineralocorticoid receptor, VEGF = vascular endothelial growth factor, ETDRS = early treatment diabetic retinopathy study, logMAR = logarithm of the minimum angle of resolution

Baseline demographics:	
Number of eyes (n)	42
Right/Left	12/30
Number of patients	39
Male/female	32/7 (82.1%/17.9%)
Mean age	48.1 ± 10.4 years (Median 49, range 25–67)
Mean symptomatic time	134 ± 133.4 weeks (median 72, range 16–518)
Previous therapy attempts (n):	
MR antagonists	33 (78.6%)
Photodynamic therapy	6 (14.3%)
Anti-VEGF	4 (9.5%)
Baseline parameters:	
Baseline visual acuity (ETDRS letter score)	79.7 ± 12.9 (35–94)
Baseline visual acuity (logMAR)	0.15 ± 0.25 (− 0.1–1)
Baseline central retinal thickness (µm)	398.4 ± 134.6 (216–902)
Baseline subfoveal choroidal thickness (µm)	430.2 ± 115.7 (150–693)
Number of eyes with choroidal thickness > 300 µm	37 (88.1%)

### Treatment data

Retreatment was performed every 3 months as long as foveal subretinal fluid persisted. During the study period, there were n = 126 laser treatments in total while the average number of treatments per eye was 3 ± 1.1 treatments (range 1–4, median 3). 14% (n = 6) eyes were treated only once, 19% (n = 8) were treated twice, 17% (n = 7) had three treatments and the remaining 45% (n = 19) had four treatments in total. The average laser power titrated with 200 µm spot size and 15 ms pulse duration was 147 ± 17.5 mW (median 150 mW, range 120–175 mW). All treatments were performed using a 400-spot macular grid pattern as described in methods. Mean spot count for titration was 6.8 ± 3.6 spots.

### Adherence to protocol

**Fig. 1:**
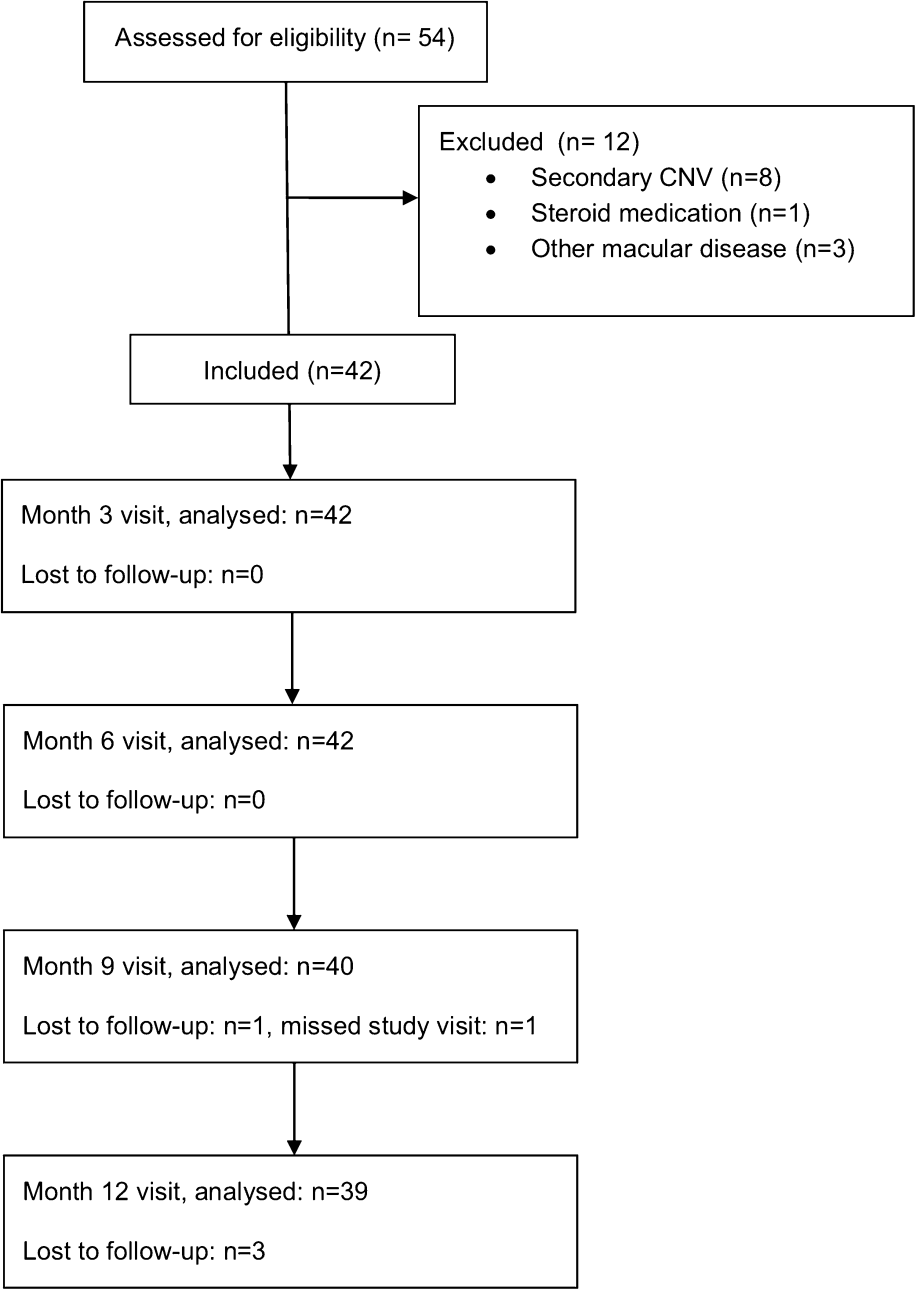
Flow diagram showing the trial population over the trial period. CNV = choroidal neovascularization

The 6-month visit was intended to be 6 months ± 30 days after the baseline treatment visit. In the study population, the mean time interval between baseline and 6-month visit was 182 ± 18.8 days (range 159–259). For the final visit, which was intended to take place at 12 months ± 30 days after baseline, the time interval was 377 ± 54 days (range 333–616). Patients who did not attend their intended study visits (7.1% (n = 3) and 17.9% (n = 7) after month 6 and 12, respectively) had to be rescheduled causing protocol deviations. Complete follow-up of all patients was available for the 6-month visit. For the 12-month visit, follow-up of 39 eyes (93%) was available (Fig. 1).

### Primary endpoint and secondary anatomical outcome measures

**Fig. 2:**
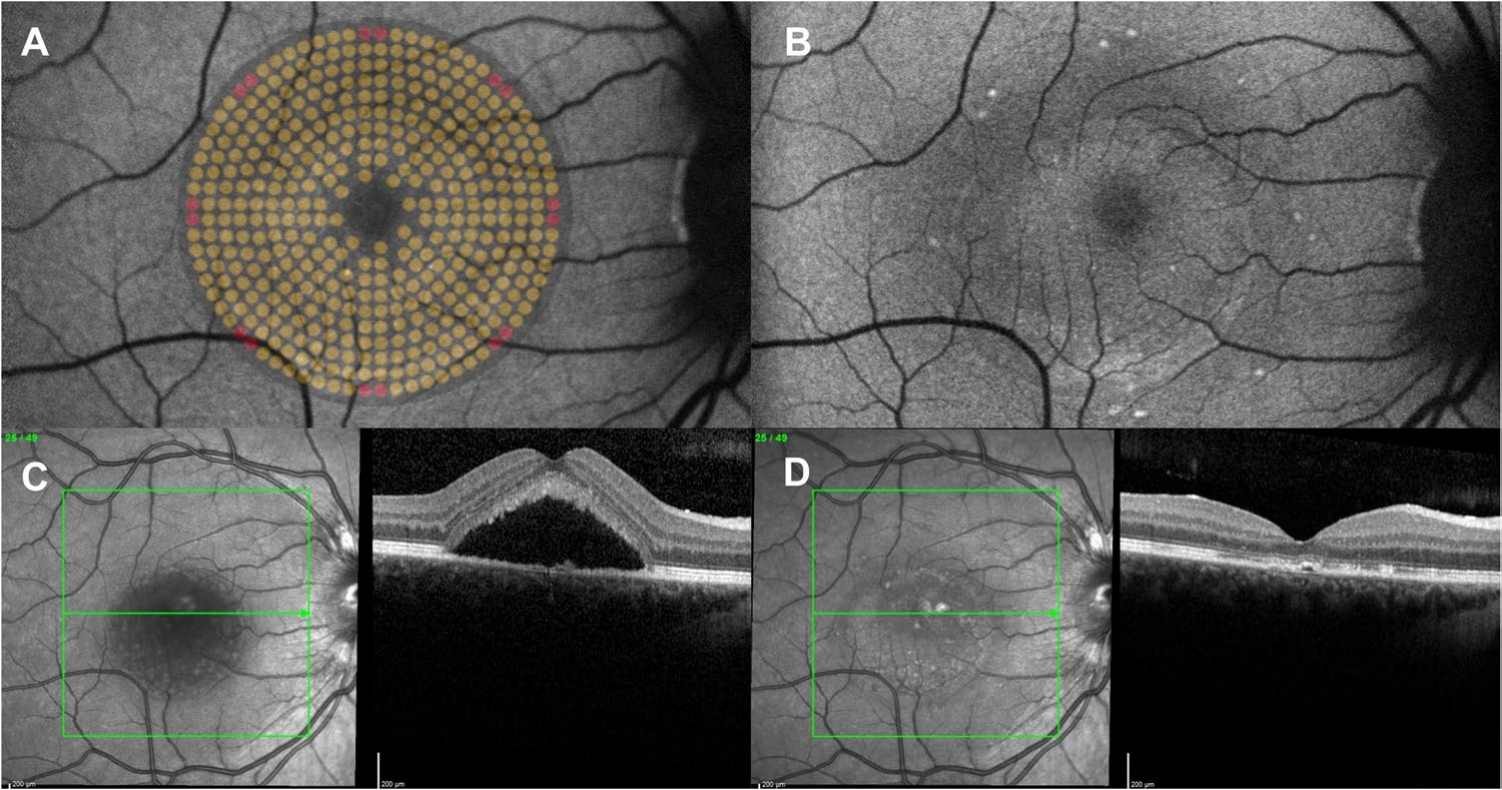
Clinical example of a 32-year-old patient. The autofluorescence images at the top row show the status before (**A**) and 3 months after (**B**) the first treatment. In (**A**), a transparent graphic overlay is indicating the actual treatment area and the treatment pattern. The red dots indicate the landmarks which are applied with the full titration energy. In (**B**) the landmarks can be seen as hyperautofluorescent dots corresponding to the graphic in (A). The images (**C**) and (**D**) show the corresponding OCT-scans to (**A**) and (**B**), respectively

The primary endpoint, defined as complete resolution of subfoveal SRF at 6 months follow-up, was reached in 18 eyes (42.9%). In 12 months follow-up, complete resolution of foveal SRF was observed in 21 eyes (53.8%). CMT decreased from 398 ± 135 µm at baseline to 291 ± 68 µm (p < 0.001) at 6 months. In the same time span, there was a subtle but significant change in SFCT (430 ± 116 µm to 419 ± 113 µm, p = 0.026). After 12 months, mean CMT was 297 ± 78 µm (n = 39) and mean SFCT was 411 ± 121 µm (n = 39). Both values did not differ significantly from the 6-month visit with p = 0.58 and p = 0.39, respectively. The change in SFCT between baseline and 12 months (429 ± 117 µm to 411 ± 121 µm) was not statistically significant (p = 0.065, n = 39) (Fig. 2).

### Secondary functional outcome measures

At 6 months, mean best corrected visual acuity (BCVA) improved by 4.9 ± 8.6 ETDRS letters (p = 0.001) compared to baseline while the average threshold of microperimetry-derived retinal sensitivity (MAIA) changed from 19.1 ± 4.7 dB to 21.3 ± 4.8 dB (p = 0.008). At 12 months follow-up, BCVA and MAIA average threshold did not change significantly compared to the 6 months results, with + 0.82 ± 5.49 ETDRS letters (p = 0.36) and + 0.10 ± 2.16 dB (p = 0.77), respectively (Table 2).

**Table 2:** Treatment data and anatomical outcomes. SRF = subretinal fluid, CMT = central macular thickness, SFCT = subfoveal choroidal thickness. Data are mean ± standard deviation

	Baseline	Month 3	Month 6	Month 9	Month 12
n	42	42	42	40	39
n (re)-treatments	42	36	25	23	/
Complete resolution of SRF (%)	0	23.8	42.9	55	53.8
CMT (µm)	398 ± 135	320 ± 79	291 ± 68	288 ± 78	297 ± 79
SFCT (µm)	430 ± 116	426 ± 114	419 ± 113	414 ± 119	411 ± 121

### Early response

There were n = 16 “early responders” after month 3. Eyes with an initial decrease in CMT by > 15% had a significantly higher proportion of complete resolution of subretinal fluid after 12 months than those without the mentioned marked decrease in CMT (80% vs. 37.5%, p = 0.01). Symptomatic time, patient age and baseline BCVA did not have a significant influence on the probability of “early response” (p = 0.16, p = 0.09 and p = 0.26, respectively), while mean baseline CMT was significantly higher in the “early response” group (500 ± 152 µm vs. 336 ± 72.5 µm, p = 0.001).

### Safety and adverse events

As described in the methods section, nonresponse to the treatment was defined as persistent subfoveal subretinal fluid (pSRF) at the study end. When analyzing the pSRF group (n = 18) individually, no statistically significant changes for the functional outcome measures BCVA and average threshold in month 6 and month 12 vs baseline were noted. There were 2 eyes (4.8%) with a loss of > 1line in BCVA.

No RPE damage as monitored by fundus autofluorescence was encountered. One patient (2.4%) received three intravitreal injections of ranibizumab between month 9 and 12 because of a subretinal lesion on OCT suspicious for choroidal neovascularization (CNV). However, CNV could not be confirmed on OCTA or FA/ICGA at the 9 or 12-month visit. Furthermore, no response to the anti-vascular endothelial growth factor (VEGF) treatment was observed. Therefore, the lesion was classified as degenerative.

## Discussion

In the present study, we prospectively evaluated a laser therapy with EpM in eyes with cCSC. In the study collective, 53.8% of the treated eyes showed a complete resolution of subfoveal subretinal fluid after 12 months. There was an overall significant decrease in central macular thickness, while BCVA and microperimetry-assessed retinal sensitivity improved.

Previously published data by Lavinsky and Palanker presenting results of 21 eyes with cCSC treated with EpM [14] showed a complete resolution of SRF in 81% of patients after 12 months of follow-up. This rate of complete resolution of SRF is substantially higher than the rate of the present study (53.8%). When looking at further results, the gain of visual acuity of 12.5 ± 4 ETDRS letters was also markedly higher while the reduction of CMT from 362 ± 11 to 283 ± 9 µm was comparable to our results (+ 6.1 ± 9.5 ETDRS letters and 406 ± 136 to 297 ± 78 µm, respectively). Subfoveal choroidal thickness decreased significantly from 349 ± 20 µm to 293 ± 17 µm in the publication of Lavinsky and Palanker while not showing a significant change in the present study (429 ± 117 µm to 411 ± 121 µm). It should be noted that the presentation of statistical dispersion differs between the publication mentioned above and the present one (mean ± standard error of the mean vs. mean ± standard deviation, respectively). The attempt to find an explanation for the divergent results leads the focus to differences in the collectives. Firstly, baseline subfoveal choroidal thickness was lower in the collective of Lavinsky and Palanker. Secondly, mean age at baseline was higher (56 ± 17 years vs. 48 ± 10 years) while mean duration of CSC symptoms before treatment was lower (11 ± 4 months vs. 31 ± 30.7 months). In summary, the collective of the present study was younger and had a longer mean disease duration as well as a higher mean subfoveal choroidal thickness than the collective of Lavinsky and Palanker. Furthermore, differences in the treatment are noticeable: The mean titrated treatment power was 147 ± 18 mW in the present study compared to 126 ± 14mW in the study of Lavinsky and Palanker. This might be attributable to ethnic differences in fundus pigmentation with one collective being recruited in Brazil and the other collective recruited in Germany. In the present study, for better standardization and comparability, all patients were treated with the same 400-spot macular grid pattern, whereas in the study of Lavinsky and Palanker, the treatment area was enlarged by adding 2 × 2 and 3 × 3 square patterns leading to a higher average spot count of 548 ± 212 spots [14].

To date, no evidence-based guidelines for the treatment of cCSC exist. When searching the literature of the past 5 years for high-level evidence, two trials in particular deserve special attention.

First, the randomized placebo-controlled multicenter trial, named “VICI trial”, designed to compare the treatment with Eplerenone, a mineralocorticoid receptor antagonist, versus placebo. The authors concluded that, in terms of visual acuity, Eplerenone was not superior to placebo [8]. This study is not comparable to the present study as no laser treatment was involved in the treatment group but it ought to be highlighted that 78.6% of the patients included in the present study had an ineffective therapy trial with MR-antagonists before inclusion which supports the findings of the VICI trial.

The second study to discuss is the “PLACE-trial” that compared the treatments half-dose PDT and “high-density subthreshold micropulse laser” (HSML) in a multicenter randomized-controlled trial setting [12]. In the PLACE-trial, the half-dose PDT group showed a complete resolution of subretinal fluid in 67.2% of the patients whereas in the HSML group only 28.8% achieved the same endpoint after the final evaluation visit which was scheduled 7 to 8 months after the initial treatment. Comparing these results with those of the present study, it could be reasoned that the performance of a laser therapy with EpM ranks between HSML and half-dose PDT. Nevertheless, it should be stressed that the comparability of these studies is limited due to several reasons. First, the HSML treatment in the PLACE trial was focused on areas of hyperfluorescence on ICG while the laser therapy in the present study was covering the central 3 mm of the macular using a fixed pattern. This leads to second, the total spot count in the HSML group of the PLACE trial was lower than in the present study which is again deferable to different treatment patterns. Third, in the HSML treatment of the PLACE-trial, the titration of laser power to “subthreshold” was limited to a reduction of laser power in cases of visible damage (discoloration) of the retina, whereas in the present study a thorough and standardized titration process was performed. Furthermore, the laser wavelengths were different with an infrared (810 nm) laser in the PLACE-trial and a yellow (577 nm) laser in the present study which leads to different energy absorption in the RPE. Interestingly, the age distribution of the patients in the PLACE-trial (48.6 ± 8.3 years in the HSML group) was similar to the age distribution of the patients in the present study (48.1 ± 10.4 years) while the mean recorded duration of disease symptoms was higher and more dispersed in the present study.

In the past decade, new imaging evidence led to the classification of CSC as part of the so-called “pachychoroid disease spectrum” [15, 16], shifting the focus of pathophysiological considerations to the choroid. Imaging hallmarks of pachychoroid disease are a thickened choroid (> 300 µm) with concomitant presence of abnormally dilated Haller layer vessels (so-called pachyvessels) that cause attenuation of the overlying Sattler layer and choriocapillaris [17]. In the presented study collective, 88.1% of the included eyes had a subfoveal choroidal thickness exceeding 300 µm. The pathophysiology of pachychoroid disease is yet unknown. A recent imaging study provided a possible explanation showing that in eyes with pachychoroid disease, the choroidal veins of the Haller layer do not respect the horizontal macular watershed and form vertical anastomoses which was attributed to possible chronic vortex vein congestion [18]. The pathological findings of pachychoroid disease implicate that choroidal changes should be targeted by the treatment. The effect of a laser treatment on the choroid is unknown. Perhaps the laser-triggered RPE processes also have a certain effect on inner choroidal layers through cellular mediators. The choroidal pathologies also provide an explanation why PDT could be an effective therapy, as PDT is known to induce a reduction of flow in choroidal perfusion seen on ICG [19]. Interestingly, PDT treatment leads to a reduction of choroidal thickness but only transient measurable effects on choroidal architecture on OCT B-scans are noticeable [20]. Supporting this, another study could not detect any changes in choroidal vascularity index, which is the ratio of luminal area versus total choroidal area on OCT B-scans, after half-dose PDT [21]. In summary, the PDT-induced temporary reduction of choroidal flow appears to allow absorption of subretinal fluid but does not lead to permanent changes of choroidal flow. In the present study, mean SFCT decreased only marginally over the observation period. The change in SFCT was statistically significant for the 6-month control versus baseline and just missed statistical significance when comparing the final visit with the baseline visit. These findings indicate that the laser therapy with EpM does not induce a marked thinning of the choroid and thus might not cause major changes in choroidal flow which leads to the consideration that the therapeutic effect on CSC has to be rather sought in the RPE.

Cellular effects of the laser therapy with EpM were extensively investigated in preclinical studies. The RPE is the primary locus of light absorption and thus the primary tissue of laser energy deposition. Instead of cell death which occurs by classical thermal laser spots, the RPE cells are solely under nonlethal thermal stress when targeted by a subthreshold (or “nondamaging”) laser beam resulting in upregulated expression of heat shock proteins (HSP) [22]. Induction of HSP helps refolding damaged proteins and HSP also have antiapoptotic functions by activation intracellular pathways, all of which is meant to enhance the vitality and fluid pumping capacity of the RPE [14]. Sublethal thermal stress may also lead to healing processes in the RPE and subsequently less permeability for the fluid originating from the dysregulated choroid. However, the exact cellular mechanisms leading to therapeutic effects of subthreshold laser therapy in cCSC remain object of further research. When discussing safety aspects of the laser therapy with EpM it has to be noted that in contrast to navigated laser systems there is no automatic eye tracking in the Topcon laser system as it is a regular slit-lamp mounted laser. As described in the methods sections, correct fixation of the patient is manually controlled by the performing ophthalmologist. Since the eye is fixed with a contact-lens, spontaneous saccades cannot occur in large angles. Small angle saccades during the application of a laser pattern should not lead to a foveal application because the central 300 µm radius are spared by the pattern.

As there is a variety of literature on micropulse laser treatments in cCSC, the laser therapy with EpM needs to be carefully distinguished from micropulse lasers. Micropulse lasers use the temporal modulation of the laser energy in order to confine the effect of heating to the light absorbing RPE and reduce the spreading of heat to the adjacent photoreceptors. In a computational simulation, this effect of confinement could be confirmed [23]. However, the authors concluded that there is no therapeutic benefit when the actual treatment goal is to avoid any cellular damage because micropulse modulation actually reduces the therapeutic window for the desired cellular effects of subthreshold (or “nondamaging”) laser therapy [23]. The key issue for subthreshold laser therapies is the titration of laser energy below the damage threshold while maintaining the therapeutic effect. In contrast to micropulse lasers, the laser used for the EpM treatment is a continuous wave laser without temporal modulation of the laser beam. In order to find a relation between the level of thermal protein denaturation (i.e. thermal damage) and laser power and duration, a mathematical model describing the temperature-dependent reaction with the Arrhenius-equation was developed and then validated in animal models [24]. The mathematical model of thermal damage was then correlated with cellular response measured as heat-shock protein expression [22]. By combining the thermal damage model with tissue response, the EpM algorithm was developed to define clinically relevant endpoints (maximizing tissue response while minimizing cellular damage) and translate them to laser energy settings [14]. Herein lies the main advantage of the laser therapy with EpM over other subthreshold laser therapy because the algorithm was validated on tissue models in preclinical studies [22, 24].

The chronic nature of CSC appears in the fact that it can be progressive by leading to complications that spur further loss of photoreceptors and subsequently loss of vision. The most common complication of cCSC is the development of secondary choroidal neovascularization with an incidence reported between 24 [3] and 39.2% [25]. Secondary CNVs in cCSC typically impose as flat irregular pigment epithelium detachments in which a type 1 CNV can be detected by OCTA [26]. Not surprisingly, in the present study, the main reason for screening failure was the detection of secondary CNV by thorough imaging. During the course of the study, no treatment related CNV was observed. Whether or not subthreshold/nondamaging laser therapies prevent or promote CNV formation is a question that cannot be answered in 12 months of follow-up due to the slow progression of cCSC. Secondary CNV in cCSC usually develop as type 1 CNV (between the RPE and Bruch membrane) which means that they are covered by vital RPE. If such a lesion evolved after a laser therapy, it could therefore not be clearly distinguished whether the treatment or the natural course of the underlying disease is causal for its development. It could be reasoned that type 2 CNV formation in the laser treatment area could be suggestive of a laser-associated damage to the RPE while type 1 CNV formation could still be attributed to progression of the underlying cCSC.

An important safety aspect of the present study is the fact that in the group of nonresponders (i.e., persistent subretinal fluid) there was no mean loss of BCVA and MAIA average threshold over the 12-month study period. This, in turn, means that deferring a half dose PDT in favor of a trial of laser therapy with EpM would not be detrimental. As a matter of fact, “early responders” to EpM who show a marked decrease of CMT (> 15%) after the first treatment are very likely to have a complete resolution of subretinal fluid (80% after one year). This could be an important predictor for treatment success and facilitate individual treatment decisions in clinical practice.

A well-known feature of CSC that requires discussion is spontaneous resolution of subretinal fluid. Spontaneous resolution of subretinal fluid is regarded as unlikely in the cases involved in this study when taking into account the long-standing persistence of subretinal fluid before inclusion. Since there is no control group in the present study, the results need to be compared with collectives reporting results of untreated patients over 12 months. The most recent and best-documented collective is the placebo arm of the aforementioned VICI trial. In the placebo arm of the VICI trial, 29.6% (16 of 54 patients) showed a complete resolution of subretinal fluid after 12 months [8]. When comparing this result with 53.8% complete resolution of subretinal fluid in the present study, it seems likely that the additional effect is a result of the laser treatment. The collectives are comparable concerning mean age (49.9 + 7.9 years in the VICI trial vs. 48 ± 10.4 years in the present study) and gender distribution (75% male in the VICI trial vs. 82.1% male patients in the present study). Interestingly, additional treatments within “usual care” for cCSC were allowed in the VICI trial. Thus, nine PDT treatments were administered in six patients of the placebo arm and one patient in the placebo arm received a subthreshold laser treatment (not further specified). This renders the placebo arm a not entirely “natural course” collective, as effective treatments were administered in a small proportion. But on the other hand, this supports the hypothesis that the better results regarding complete resolution of subretinal fluid in the present study could be interpreted as a consequence of the intervention.

The strengths of the presented study include its prospective design, the thorough multimodal imaging and the involvement of microperimetry as a functional outcome. This study so far presents the largest cohort of patients treated with a laser therapy with EpM for cCSC. A main limitation is the absence of a control group. Moreover, an actual strength of the study, the inclusion of very chronic cases with multiple prior treatment trials, could also be interpreted as a limitation, as it leads to a high heterogeneity of the study collective and makes treatment effects less comparable. A further limitation was the adherence to the protocol: some patients did not show good adherence to the provided time slots and therefore caused outliers in the timetable. Based on the modified intention to treat approach, data was analyzed for the respective study visits regardless of correct timing. Nevertheless, the mean interval between study visits was as intended by the protocol.

In conclusion, this study presents a prospective clinical evaluation of a laser therapy with EpM with the so far largest cohort for this technology and the results show that a complete resolution of subretinal fluid is achievable in a considerable number of patients with longstanding CSC as well as a significant improvement of function. The combination of a standardized visible titration with an algorithm that adjusts the laser parameters facilitates treatment safety and reproducibility. The provided results may serve as a basis for power calculation of further controlled studies.
